# Implications of variability in triceps surae muscle volumes on peak lower limb muscle forces during human walking

**DOI:** 10.1371/journal.pone.0320516

**Published:** 2025-03-28

**Authors:** Christine M. Harper, Adam D. Sylvester, Patricia Ann Kramer

**Affiliations:** 1 Department of Anthropology, University of Washington, Seattle, Washington, United States of America; 2 Department of Biomedical Sciences, Cooper Medical School of Rowan University, Camden, New Jersey, United States of America; 3 Center for Functional Anatomy and Evolution, The Johns Hopkins University School of Medicine, Baltimore, Maryland, United States of America; University College Dublin, Ireland

## Abstract

Musculoskeletal modeling can be used to estimate forces during locomotion. These models, however, are dependent on underlying assumptions about the model inputs, such as muscle volumes and fiber lengths, to calculate muscle forces. Triceps surae (gastrocnemius medialis, gastrocnemius lateralis, soleus) muscle volume distributions vary among humans. Here we quantify how this muscle volume variation impacts maximum estimated lower limb muscle forces during the braking and propulsive phases of the stance phase of walking. Three triceps surae muscle volume distributions (AnyBody Modeling System standard cadaver [MS], average of 21 cadavers [C], average of 21 young, healthy adults [YHA]) were evaluated in a standard musculoskeletal model using the kinetic and kinematic data of 10 healthy individuals at three walking velocities. Maximum muscle forces were calculated using inverse dynamics and an algorithm to solve the muscle redundancy problem in the AnyBody Modeling System. Repeated measure ANOVAs were used to test for significant differences among the three muscle distribution configurations for each muscle/muscle group at each velocity. Triceps surae muscle volume distribution significantly affects gastrocnemius lateralis and soleus maximum muscle forces for both braking and propulsion at all three velocities (p < 0.001), with relatively larger muscle volumes typically producing relatively larger muscle forces. There was no significant difference in gastrocnemius medialis maximum force among configurations (p > 0.124) except at the self-selected spontaneous velocity during braking. Significant differences exist at some velocities for the hamstrings and gluteus maximus during braking (p < 0.046) and the other plantarflexors, dorsiflexors, evertors, hamstrings, quadriceps, sartorius, and gluteus maximus during propulsion (p < 0.042). Muscle volumes used in musculoskeletal models impact estimated muscle forces of both the muscles of interest and other muscles in the biomechanical chain. This is consistent with recent analyses demonstrating that input values can substantially impact results and suggests individualized muscle parameters may be needed depending on the research question.

## Introduction

Musculoskeletal modeling is a powerful engineering approach to estimate forces during locomotion and has applications to wide range of fields including medicine, anthropology, and biology [1]. These models can be used to estimate internal (e.g., muscle and joint reaction) forces based on individualized kinetic and kinematic data, which allows for both investigations of human variation during movement and the development of patient specific models [1]. Musculoskeletal models are particularly relevant for investigating muscle variation as empirically measuring muscle forces in living individuals is exceptionally difficult because it requires the surgical implantation of sensors [2–3]. As with all models, however, musculoskeletal models are dependent on underlying assumptions and input parameters, such as muscle properties, including muscle volume, physiological cross-sectional area (PCSA), muscle fiber length, and pennation angle [4]. It is thus important to understand how variation in such model inputs and assumptions affect model outputs.

Considering the implications of muscle parameters on muscle function during locomotion is particularly important for the triceps surae muscle complex due to its role as the primary propulsive driver during walking [5]. The triceps surae muscle complex consists of the gastrocnemius lateralis, gastrocnemius medialis, and soleus, which all attach distally to the calcaneal tuberosity via the Achilles tendon and are ankle plantarflexors. Gastrocnemius medialis and lateralis attach proximally on the medial and lateral femoral condyles, respectively, and thus additionally act as knee flexors, while soleus proximally attaches to the tibia and fibula and solely acts on the ankle.

Triceps surae muscle properties, including volume, PCSA, muscle fiber length, and pennation angle have thus been extensively quantified in both cadaveric donors by directly measuring the dissected muscles [6–8] and in living humans using magnetic resonance imaging (MRI), diffusor tensor imaging (DTI), and ultrasound [9–21]. These analyses have found that muscle volumes of the gastrocnemius medialis, gastrocnemius lateralis, and soleus, as well as the distribution of volume across the three muscles, vary substantially among individuals (Table 1). Understanding the functional implications of these differences is critical as muscle volume is used to determine PCSA, which directly impacts the amount of force that can be produced [15].

**Table 1:** Variation in average triceps surae muscle volumes/distributions across the literature.

Subjects	Age (years)^1^	Sample Notes	Imaging/Approach	Gastrocnemius Lateralis Volume (mL)^2^	Gastrocnemius Medialis Volume (mL)^2^	Soleus Volume (mL)^2^	Citation
13M	29 (6)	University Staff/Students	MRI and Ultrasound	146 (16.08%)	285 (31.39%)	477 (52.53%)	9
5M, 5F	27 (4)	Non-Professional Athletes	MRI and Diffusion Tensor Imaging	128 (15.63%)	230 (28.08%)	461 (56.29%)	11
10M, 10F	26 (6)	–	MRI	137.7 (17.05%)	249.7 (30.92%)	420.1 (52.03%)	12
8F, 7M	18 (0.6)	NCAA Sprinters	MRI	161 (17.89%)	273 (30.33%)	466 (51.78%)	14
8F, 16M	25.5 (11.1)	Physically Active	MRI	150 (17.74%)	257.4 (30.44%)	438.2 (51.82%)	15
7M	23.9 (2)	–	MRI	130.3 (16.14%)	247 (30.59%)	430.2 (53.27%)	16
6F, 8M	26 (4)	Active	PET/MR Scanner	175.1 (18.59%)	291.6 (30.96%)	475.2 (50.45%)	17
4F, 3M	66 (5)	Healthy, Daily Activity	PET/MR Scanner	151.9 (19.50%)	231.1 (29.66%)	396.1 (50.84%)	17
4M, 4F	31 (6)	–	MRI and Diffusion Tensor Imaging	140 (16.55%)	256.9 (30.36%)	449.2 (53.09%)	10
11F	69 (7)	Post-Menopausal	MRI	75.4 (13.09%)	160.6 (27.90%)	339.6 (55.51%)	18
15M	25.3 (4.5)	Physically Active	MRI	177.8 (17.76%)	303.2 (30.29%)	520.1 (51.95%)	19
12M	73.8 (4.4)	Physically Active	MRI	130.5 (16.81%)	215 (27.68%)	431.1 (55.51%)	19
8F, 13M	24.6 (4.3)	–	MRI	150.1 (18.80%)	243.7 (30.52%)	404.6 (50.68%)	21
6F, 9M	70.4 (2.4)	–	MRI	138.3 (17.54%)	219.5 (27.84%)	430.7 (54.62%)	21
9M, 12F	83 (9)	Formaldehyde fixed cadavers	Direct measurement after removal	62.2 (13.77%)	113.5 (25.14%)	275.8 (61.09%)	8
11M, 1F	32.6 (8.2)	–	MRI	140.8 (16.11%)	243.7 (27.90%)	489.1 (55.99%)	13

^1^Average age (standard deviation);

^2^Avergae muscle volume (percentage of total triceps surae muscle volume).

Calculating forces for individual muscles using musculoskeletal modeling can be a challenge because of the muscle redundancy problem (i.e., how to apportion net joint moments among multiple muscles that act together to produce the same motion at a joint) [4]. Algorithms employed by musculoskeletal models to solve the muscle redundancy problem utilize muscle parameters, such as muscle volume, in their calculations to determine muscle activation levels via a cost or allocation algorithm[4]. It is thus essential to understand the sensitivity of musculoskeletal models to variation in muscle parameters, specifically the relationship between muscle volume and estimated muscle force, as these are commonly used to understand lower limb biomechanics and estimate forces acting on bone [22]. Muscle volume is particularly important to investigate in the AnyBody Modeling System (the musculoskeletal model employed in this study) because it, along with fiber length and pennation angle, is used to determine PCSA [4].

Here we investigate how variation in the distribution of muscle volume across the triceps surae muscle complex impacts forces produced by the gastrocnemius medialis, gastrocnemius lateralis, and soleus muscles, as well as the other lower limb muscles, during walking at multiple velocities. More specifically, we investigate three muscle distribution configurations: the standard cadaveric model muscle volumes used in the AnyBody Modeling System (MS configuration) [6], the average muscle volumes for 21 cadaveric donors (C configuration) that are commonly used by other musculoskeletal modeling systems (e.g., OpenSim) [8], and the average of 21 young, healthy living individuals from the literature (YHA configuration) [21] (Table 2). Although there are many studies investigating triceps surae muscle volumes in living individuals, data from Pinel and colleagues [21] was chosen for the YHA configuration because it has the highest gastrocnemius muscle volume relative to total triceps surae volume and soleus was not measured in multiple parts (i.e., whole soleus muscle volume was measured, rather than subsections of the muscle). The MS cadaver exhibits the highest soleus volume relative to total triceps surae volume, allowing us to examine a range of variation across the three muscle volume distributions. We chose to use published data on triceps surae muscle volumes, rather than collect new data, as muscle data from the literature are typically used in musculoskeletal models. This approach thus allows us to investigate how sensitive musculoskeletal models are to muscle input parameters and determine if individualized muscle parameters are necessary. Multiple velocities were included because of the known impacts of velocity on walking kinetics and kinematics. We hypothesize that changes in muscle volume will impact the maximum force produced by that muscle during straight-path walking at multiple velocities and predict that the soleus muscle force will be relatively highest in the MS configuration and that gastrocnemius muscle force will be relatively highest in the YHA configuration.

**Table 2:** Triceps surae muscle volume configurations and adjusted baseline musculoskeletal model muscle volumes investigated in this study.

Configuration	Gastrocnemius Lateralis Volume (mL)^1^	Gastrocnemius Medialis Volume (mL)^1^	Soleus Volume (mL)^1^
Model-Standard Cadaver (MS) [6]	136.36 (11.43%)	263.26 (22.06%)	792.91 (66.49%)
Average of 21 Cadavers (C) [8]	164.29 (13.77%)	299.78 (25.14%)	728.46 (61.09%)
Average of 21 Young, Healthy Adults (YHA) [21]	224.20 (18.80%)	364.00 (30.52%)	604.33 (50.68%)

^1^Adjusted baseline muscle volume based on different muscle configurations (percentage of total triceps surae muscle volume). The adjusted muscle volumes were calculated by taking the total muscle volume of the baseline musculoskeletal model and reapportioning those values based on the percentage of volume represented by each of the configurations.

We also hypothesize that changing triceps surae muscle volume distributions will impact the maximum muscle forces produced by other lower limb muscles/muscle groups. We predict that when gastrocnemius medialis and gastrocnemius lateralis muscle volume (as in the YHA configuration) is relatively larger, the quadriceps muscle force will be larger to compensate for the relative larger knee flexion moments at the knee joint. Similarly, we predict that the hamstrings muscle forces will be relatively higher when soleus muscle volume is relatively higher (as in the MS configuration) to compensate for relatively smaller knee flexion moments.

## Materials and methods

### Subjects

Existing motion capture (52 optical markers) and ground reaction force data for 10 healthy, adult subjects (five males and five females; from Schreiber & Moissenet [23]) were utilized in this study (Table 3). Data from trials representing three velocities from the original study were included: slow normal (0.8-1.2 m/s, C3), self-selected spontaneous (0.9-1.5 m/s, C4), and self-selected fast (1.3-2.4 m/s, C5) [23]. Each velocity was represented by three to five trials. Any trials for which the right and left foot did not fully contact the single force plate were discarded.

**Table 3:** Subjects included in this study (from Schreiber & Moissenet [23]).

Subject	Gender	Age (years)	Height (m)	Body Mass (kg)
2014001	M	31	1.66	67.0
2014013	F	26	1.70	61.3
2014014	M	29	1.80	92.0
2014019	F	26	1.76	73.8
2014031	M	21	1.77	67.2
2014040	F	19	1.55	56.5
2014048	F	40	1.64	61.5
2014051	M	25	1.91	88.0
2015016	F	46	1.69	76.0
2015030	M	24	1.87	86.0

### Baseline musculoskeletal model

The baseline musculoskeletal model utilized in this study was the AnyBody Modeling System (v7.4, AnyBody Technology, Denmark) ADL Gait (beta) Fullbody MoCap model hosted on the AnyBody Managed Model Repository (AMMR v2.4.2) created following [1,4,24,25]. This is a full body motion-capture driven model of human gait containing segments representing the head, trunk, and both the right and left upper and lower limbs [26]. The data on muscle architecture in the baseline model are based on a single cadaveric specimen [6] (Table 2). Motion of all segments in the musculoskeletal model is driven by the motion capture data from Schreiber & Moissennet [23].

Each lower limb is comprised of six individual segments in the model, including the pelvis, thigh, patella, shank, talus, and foot. The lower limb joints allow for a total of six degrees of freedom, including three rotations at the hip, and one at the knee (flexion/extension), ankle (plantarflexion/dorsiflexion), and subtalar (inversions/eversion) joints. Each lower limb includes forty-one muscles, which are composed of 169 muscle elements. Gastrocnemius medialis and lateralis are each represented by a single muscle element with via points to follow the muscle’s anatomical path over the distal femur to the calcaneus. Soleus is represented by six muscle elements to account for the distributed proximal attachment of the anatomical muscle across the tibia and fibula. Total muscle volume for soleus is evenly divided over the six muscle elements.

Initial marker driver positions and segment parameters that produce the best fit for overall motion for each subject were determined previously [24]. In brief, segment parameters, such as pelvic width and femoral length, were determined using the first self-selected spontaneous velocity trial and analyzed using the Parameter Identification routine in the AnyBody Modeling System [24]. As a result, each subject has an individually sized model based on their anthropometrics and motions. Muscle forces during braking and propulsion for these data were then calculated in the AnyBody Modeling System using inverse dynamics, which includes a built-in muscle recruitment algorithm to solve the muscle redundancy problem.

### New muscle parameter models

Additional musculoskeletal models with revised triceps surae muscle volumes were generated for each subject. To adjust the muscle volumes, the total volume of the triceps surae muscle complex in the cadaver standard to the AnyBody Modeling System (based on published values for the subject) [6] was first determined. The ratio of each muscle volume (gastrocnemius medialis, gastrocnemius lateralis, soleus) relative to total muscle volume for the data representing the YHA and C configurations was then calculated [8,21]. For each of the three muscles, the ratio was then multiplied by the total triceps surae muscle volume of the model-standard cadaver. Each individual trial was then simulated using these different muscle configurations for all subjects. This approach was taken, rather than changing the individual muscle volumes based on gross published values, to ensure that we were evaluating how differences in triceps surae muscle distributions impact estimated maximum muscle forces, rather than the implications of having an overall larger triceps surae muscle complex. Relative triceps surae muscle volume distributions and adjusted muscle volumes are provided in Table 2.

### Statistical analyses

Muscle forces were extracted from AnyBody results files using custom MATLAB (Mathworks, Inc., Natick, MA) programs. Midstance for each of the two available force plates was established based on gait events (i.e., heel strike, toe off; determined by Schreiber & Moissenet [23]) following Kramer & Sylvester [24]. Midstance on force plate 1 to midstance on force plate 2 was used to represent a single step [24]. More specifically, the propulsion phase of a step was represented by midstance to toe off on force plate 1, while the braking phase was represented by heel strike to midstance on force plate 2 for all analyses. All muscle force data (or muscle group force data; described below) was then interpolated at 1% increments of total step (braking +  propulsion). The average muscle force profile was then determined for each subject at each velocity for all three muscle configurations. The maximum muscle force during both braking and propulsion was extracted for each subject, at each velocity, for all three parameter models and retained for further analysis.

Variation in average maximum muscle forces (i.e., the average of each subject’s maximum muscle force across trials at a given velocity) during braking and propulsion were investigated for 13 lower limb muscles (or groups of muscles) (Table 4). Maximum muscle forces typically occur at a similar percent stance for each muscle configuration and all individuals for each muscle/muscle group (S1 Figure). Muscle force was determined by summing the calculated forces of the muscle elements within the group (see Table 4 for a description of which muscles were included in the different groups). In the AnyBody Modeling System, muscle element forces reflect muscle activations. We chose to present muscle forces rather than activations for ease of understanding, even though we recognize that muscle element forces are vectors and should not normally be summed. It is important to note, that when statistically analyzed, there are no differences in results if muscle forces or activations are used. Average maximum muscles forces were normalized by subject body weight. To test for differences in average maximum muscle forces across the three triceps surae volume distributions, repeated measure analyses of variance (ANOVAs) were run in MATLAB v. 2022b (Mathworks, Inc., Natick, MA). A Greenhouse-Geiser correction was utilized in the repeated measure ANOVA as the data did not meet expectations of sphericity based on Mauchly’s test (*p < *0.05 for all tests) [27–28]. Braking and propulsion were analyzed separately for each muscle/muscle group and the three velocities were individually analyzed within the two stance phases. Each velocity category was analyzed separately to avoid conflating variation in muscle forces impacted by velocity with those driven by differences among the muscle configurations. The alpha value for statistical significance was set at 0.05. Variation in muscle forces across the three configurations during both braking and propulsion was visualized for each subject at each velocity.

**Table 4:** Muscles/muscle groups for which average maximum muscle forces were evaluated.

Muscle/Muscle Group	Muscles Included (if multiple)
Gastrocnemius Lateralis	–
Gastrocnemius Medialis	–
Soleus	–
Other Plantarflexors	Tibialis Posterior Flexor Hallucis Longus Flexor Digitorum Longus
Dorsiflexors	Tibialis Anterior Extensor Hallucis Longus Extensor Digitorum Longus
Invertors	Tibialis Anterior Tibialis Posterior Extensor Hallucis Longus Flexor Hallucis Longus Flexor Digitorum Longus
Evertors	Fibularis Longus Fibularis Brevis
Gracilis	–
Other Hip Adductors	Adductor Longus Adductor Magnus
Hamstrings	Biceps Femoris Semimembranosus Semitendinosus
Quadriceps	Vastus Lateralis Vastus Intermedius Vastus Medialis Rectus Femoris
Sartorius	–
Gluteus Maximus	–

## Results

Summary statistics for all muscle/muscle group average maximum forces can be seen in Tables 5-7. (See supporting data file.)

**Table 5:** Summary statistics of average maximum forces (N) normalized by subject body weights (N) during braking and propulsion (C3; slow normal velocity).

Muscle/Muscle Group	Braking			Propulsion		
	MS	C	YHA	MS	C	YHA
Gastrocnemius Lateralis	0.36 (0.13)	0.41 (0.14)	0.54 (0.18)	0.73 (0.13)	0.85 (0.17)	1.21 (0.25)
Gastrocnemius Medialis	0.95 (0.43)	0.96 (0.42)	0.99 (0.43)	1.51 (0.54)	1.55 (0.58)	1.64 (0.67)
Soleus	0.69 (0.24)	0.54 (0.15)	0.36 (0.11)	1.57 (0.35)	1.20 (0.28)	0.80 (0.18)
Other Plantarflexors	0.11 (0.07)	0.11 (0.09)	0.11 (0.09)	0.11 (0.16)	0.12 (0.16)	0.09 (0.14)
Dorsiflexors	0.78 (0.31)	0.68 (0.31)	0.68 (0.31)	0.09 (0.03)	0.10 (0.03)	0.10 (0.03)
Invertors	0.66 (0.25)	0.57 (0.26)	0.57 (0.26)	0.14 (0.15)	0.15 (0.15)	0.13 (0.14)
Evertors	0.85 (0.36)	0.77 (0.36)	0.76 (0.36)	1.02 (0.30)	0.90 (0.31)	0.85 (0.31)
Gracilis	0.01 (0.00)	0.01 (0.00)	0.01 (0.00)	0.04 (0.01)	0.04 (0.01)	0.04 (0.01)
Other Hip Adductors	0.17 (0.04)	0.18 (0.04)	0.18 (0.04)	0.25 (0.06)	0.30 (0.08)	0.30 (0.08)
Hamstrings	0.68 (0.31)	0.70 (0.31)	0.70 (0.31)	0.19 (0.14)	0.16 (0.12)	0.13 (0.11)
Quadriceps	0.61 (0.28)	0.66 (0.27)	0.67 (0.27)	0.85 (0.19)	0.89 (0.19)	0.98 (0.21)
Sartorius	0.06 (0.03)	0.05 (0.03)	0.04 (0.02)	0.15 (0.05)	0.17 (0.06)	0.17 (0.06)
Gluteus Maximus	0.29 (0.11)	0.22 (0.07)	0.22 (0.07)	0.05 (0.04)	0.03 (0.04)	0.03 (0.05)

Means presented with standard deviations (in parentheses). MS = Model-Standard Cadaver; C = Cadavers; YHA = Young, Healthy Adults (see Table 2).

**Table 7:** Summary statistics of average maximum forces (N) normalized by subject body weights (N) during braking and propulsion (C5; self-selected fast velocity).

Muscle/Muscle Group	Braking			Propulsion		
	MS	C	YHA	MS	C	YHA
Gastrocnemius Lateralis	0.24 (0.11)	0.28 (0.12)	0.38 (0.16)	0.87 (0.26)	1.07 (0.25)	1.53 (0.37)
Gastrocnemius Medialis	0.61 (0.29)	0.62 (0.29)	0.66 (0.31)	1.34 (0.74)	1.36 (0.73)	1.36 (0.81)
Soleus	0.47 (0.19)	0.38 (0.16)	0.24 (0.10)	1.54 (0.62)	1.23 (0.44)	0.82 (0.29)
Other Plantarflexors	0.06 (0.05)	0.07 (0.06)	0.06 (0.05)	0.02 (0.04)	0.02 (0.04)	0.02 (0.03)
Dorsiflexors	1.48 (0.62)	1.21 (0.51)	1.21 (0.51)	0.32 (0.32)	0.36 (0.37)	0.36 (0.37)
Invertors	1.22 (0.49)	1.00 (0.42)	1.00 (0.42)	0.24 (0.27)	0.28 (0.30)	0.27 (0.31)
Evertors	1.69 (0.71)	1.44 (0.63)	1.44 (0.63)	1.60 (0.41)	1.52 (0.42)	1.49 (0.44)
Gracilis	0.02 (0.01)	0.02 (0.01)	0.02 (0.01)	0.08 (0.02)	0.08 (0.02)	0.08 (0.02)
Other Hip Adductors	0.35 (0.06)	0.35 (0.06)	0.35 (0.06)	0.51 (0.11)	0.64 (0.18)	0.64 (0.18)
Hamstrings	0.79 (0.35)	0.81 (0.35)	0.81 (0.35)	0.39 (0.29)	0.35 (0.25)	0.32 (0.25)
Quadriceps	1.57 (0.46)	1.62 (0.53)	1.62 (0.54)	1.45 (0.41)	1.54 (0.44)	1.62 (0.44)
Sartorius	0.08 (0.03)	0.08 (0.03)	0.07 (0.03)	0.22 (0.05)	0.25 (0.06)	0.24 (0.06)
Gluteus Maximus	0.49 (0.18)	0.40 (0.15)	0.40 (0.15)	0.12 (0.10)	0.09 (0.11)	0.09 (0.11)

Means presented with standard deviations (in parentheses). MS = Model-Standard Cadaver; C = Cadavers; YHA = Young, Healthy Adults (see Table 2).

### Triceps surae muscles

**Fig 1:**
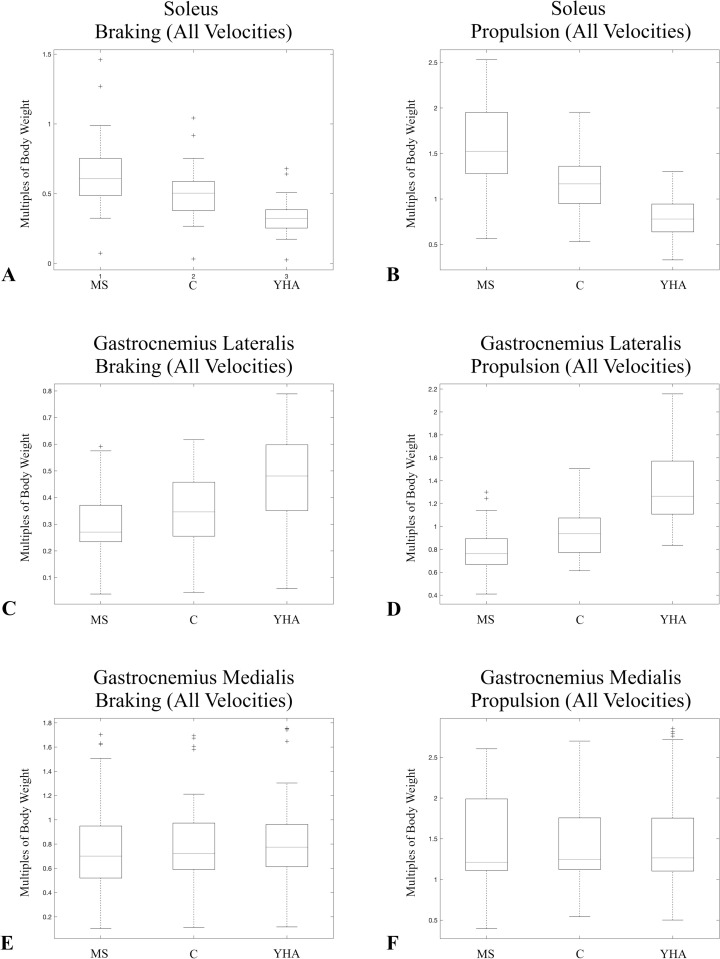
Boxplots of the average maximum muscle forces normalized by subject body weights for the triceps surae muscles during braking and propulsion at all three velocities for different muscle configurations. There were significant differences among the three muscle configurations during braking and propulsion at all three velocities for both soleus and gastrocnemius lateralis. There was only a significant difference for gastrocnemius medialis at the self-selected spontaneous velocity (C4) during braking. Soleus (A&B), gastrocnemius lateralis (C&D), and gastrocnemius medialis (E&F).

Soleus significantly differs among the three configurations for both braking and propulsion at all three velocities (Table 8). For both braking and propulsion, the MS configuration, which has the relatively largest soleus muscle volume, produces the highest soleus muscle force, while the YHA configuration (relative smallest soleus muscle volume) has the relatively lowest soleus muscle force (Fig 1A-B).

**Table 8:** Repeated Measure ANOVA results comparing the three muscle configurations for each velocity.

Muscle/Muscle Group	Braking			Propulsion		
	C3	C4	C5	C3	C4	C5
Gastrocnemius Lateralis	**<0.001**	**<0.001**	**<0.001**	**<0.001**	**<0.001**	**<0.001**
Gastrocnemius Medialis	0.225	**<0.001**	0.202	0.124	0.417	0.578
Soleus	**<0.001**	**<0.001**	**<0.001**	**<0.001**	**<0.001**	**<0.001**
Other Plantarflexors	0.543	0.649	0.655	**0.038**	0.199	0.470
Dorsiflexors	0.142	0.135	0.332	**0.011**	0.115	0.113
Invertors	0.136	0.126	0.334	0.207	0.375	0.781
Evertors	0.238	0.203	0.495	**0.002**	**0.001**	**0.018**
Gracilis	0.994	0.740	0.739	0.553	0.537	0.295
Other Hip Adductors	0.115	0.112	0.439	0.066	0.077	0.054
Hamstrings	0.211	**0.032**	0.224	**0.012**	**0.016**	0.158
Quadriceps	0.171	0.179	0.492	**0.004**	**0.014**	**0.040**
Sartorius	0.067	0.268	0.076	**0.029**	**0.034**	0.057
Gluteus Maximus	**0.025**	**0.014**	**0.046**	**0.042**	0.086	0.062

C3 = slow normal velocity; C4 = self-selected spontaneous velocity; C5 = self-selected fast velocity.

* Bold values indicate statistically significant (*p* < 0.05).

Gastrocnemius lateralis significantly differs among the three configurations for both braking and propulsion at all three velocities (Table 8). For both braking and propulsion, the YHA configuration, which has the relative highest gastrocnemius lateralis muscle volume, produces the relatively highest gastrocnemius lateralis force, while the MS configuration has the relatively lowest (Fig 1C-D).

There are no significant differences in average maximum gastrocnemius medialis force among the three configurations during propulsion nor the slow normal and self-selected fast velocities during braking (Table 8, Fig 1E-F). There is a significant difference during the self-selected spontaneous velocity (p < 0.001; Table 8), with the YHA configuration producing the highest gastrocnemius medialis force and the MS configuration the relative lowest (Fig 1E; Table 6).

**Table 6:** Summary statistics of average maximum forces (N) normalized by subject body weights (N) during braking and propulsion (C4; self-selected spontaneous velocity).

Muscle/Muscle Group	Braking			Propulsion		
	MS	C	YHA	MS	C	YHA
Gastrocnemius Lateralis	0.33 (0.13)	0.38 (0.13)	0.51 (0.17)	0.81 (0.18)	0.95 (0.21)	1.35 (0.32)
Gastrocnemius Medialis	0.85 (0.41)	0.88 (0.41)	0.93 (0.44)	1.52 (0.56)	1.56 (0.60)	1.60 (0.67)
Soleus	0.77 (0.31)	0.60 (0.21)	0.39 (0.14)	1.63 (0.41)	1.24 (0.30)	0.83 (0.20)
Other Plantarflexors	0.09 (0.07)	0.10 (0.08)	0.09 (0.07)	0.08 (0.16)	0.08 (0.14)	0.06 (0.13)
Dorsiflexors	1.02 (0.45)	0.85 (0.36)	0.85 (0.36)	0.11 (0.03)	0.12 (0.03)	0.12 (0.03)
Invertors	0.86 (0.36)	0.72 (0.29)	0.72 (0.29)	0.12 (0.14)	0.12 (0.13)	0.11 (0.11)
Evertors	1.09 (0.57)	0.95 (0.46)	0.94 (0.46)	1.15 (0.36)	1.02 (0.34)	0.97 (0.35)
Gracilis	0.01 (0.01)	0.01 (0.01)	0.01 (0.01)	0.05 (0.01)	0.05 (0.01)	0.04 (0.01)
Other Hip Adductors	0.22 (0.05)	0.23 (0.05)	0.23 (0.05)	0.32 (0.09)	0.38 (0.13)	0.38 (0.13)
Hamstrings	0.71 (0.29)	0.73 (0.30)	0.73 (0.30)	0.18 (0.10)	0.15 (0.08)	0.13 (0.07)
Quadriceps	0.85 (0.44)	0.90 (0.47)	0.90 (0.47)	0.98 (0.24)	1.03 (0.28)	1.12 (0.30)
Sartorius	0.05 (0.02)	0.05 (0.02)	0.04 (0.02)	0.17 (0.05)	0.19 (0.07)	0.18 (0.06)
Gluteus Maximus	0.34 (0.13)	0.26 (0.08)	0.26 (0.08)	0.05 (0.04)	0.03 (0.04)	0.03 (0.04)

Means presented with standard deviations (in parentheses). MS = Model-Standard Cadaver; C = Cadavers; YHA = Young, Healthy Adults (see Table 2).

### Other muscles/muscle groups

**Fig 2:**
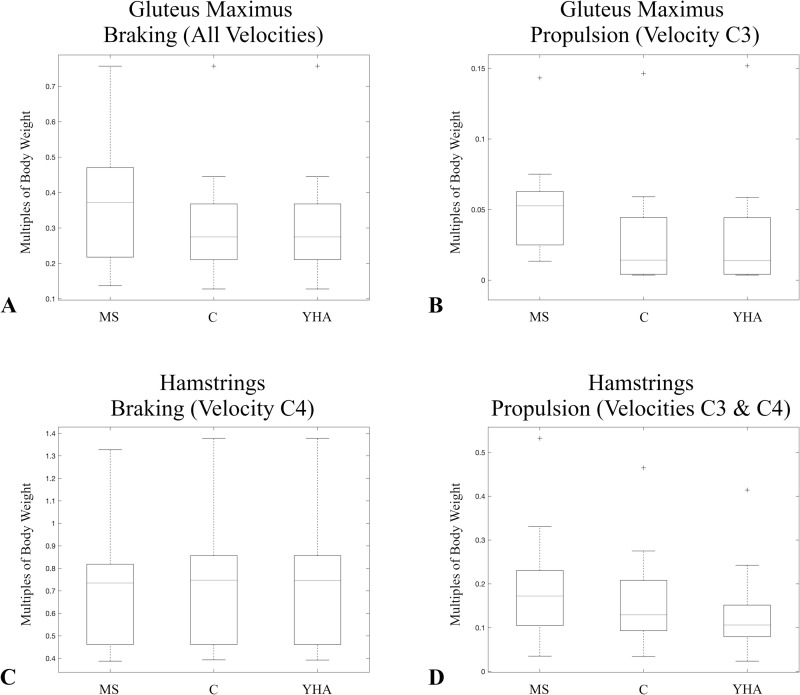
Boxplots of the average maximum muscle forces normalized by subject body weights of other muscles/muscle groups that significantly differed among the configurations during both braking and propulsion. Only those velocities for which there was a significant difference are presented. Velocities were combined in a single boxplot when there were significant differences for multiple velocities for a given muscle/muscle group and there was a consistent pattern in which configuration produced the largest muscle force. Gluteus maximus (A&B) and hamstrings (C&D). Velocity C3 is slow normal and velocity C4 is self-selected spontaneous.

During braking there are significant differences among the three configurations in the average maximum muscle forces of gluteus maximus at all three velocities and the hamstrings at the self-selected spontaneous velocity (Table 8). The MS configuration generally produces relatively higher gluteus maximus maximum muscle forces than the other two configurations (Fig 2A). For the hamstrings, the significant difference at the self-selected spontaneous velocity is driven by relatively higher forces in the YHA and C configurations compared to MS (Fig 2C).

**Fig 3:**
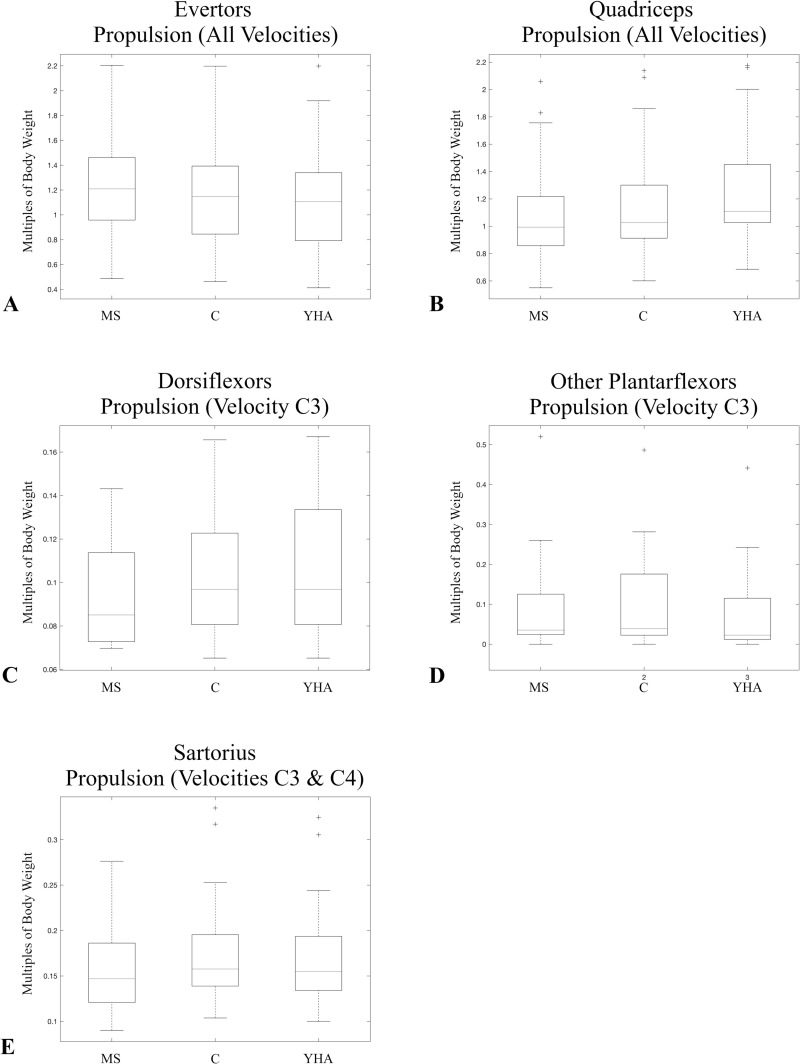
Boxplots of the average maximum muscle forces normalized by subject body weights of other muscles/muscle groups that significantly differed among the configurations during propulsion. Only those velocities for which there was a significant difference are presented. Velocities were combined in a single boxplot when there were significant differences for multiple velocities for a given muscle/muscle group and there was a consistent pattern in which configuration produced the largest muscle force. Evertors (A), quadriceps (B), dorsiflexors (C), other plantarflexors (D), and sartorius (E). Velocity C3 is slow normal and velocity C4 is self-selected normal.

During propulsion there are significant differences among the three configurations in the average maximum muscle forces of the evertors and quadriceps during all three velocities (Table 8). There are also significant differences in the ankle dorsiflexors, other plantarflexors, and gluteus maximus at the slow normal velocity, and the hamstrings and sartorius at the slow normal and self-selected spontaneous velocities (Table 8). For the evertors, the MS configuration generally has a relatively higher average muscle force than other two configurations (Fig 3A). For the quadriceps, the YHA configuration has the relative highest force, while the MS configuration has the relative lowest (Fig 3B). For the dorsiflexors, the YHA and C configurations generally have a relatively higher force than the MS configuration (Fig 3C). For the other plantarflexors (at the slow normal velocity), the MS configuration has the highest muscle force, while the YHA configuration has the relative lowest (Fig 3D). The same progression is seen for the hamstrings at slow normal and self-selected spontaneous velocities (Fig 2D). At the slow normal velocity, the MS configuration generally produces a higher average gluteus maximus muscle force than either the C or YHA configurations (Fig 2B). For sartorius, both the YHA and C configurations generally produce relatively higher force than the MS configuration (Fig 3E).

## Discussion

### Triceps surae muscles

Maximum gastrocnemius lateralis and soleus muscle forces during both braking and propulsion are significantly impacted by triceps surae muscle volume distribution, consistent with our hypothesis. For both muscles, the estimated force is relatively higher when muscle volume is relatively larger. This is consistent with the well-accepted understanding that, in vivo, muscle volume, due to its role in determining PCSA, impacts the amount of force a given muscle can generate [13,15,29]. Muscle force differences impact the forces experienced by bony elements and differences of this magnitude could be important to consider for patient-specific models. In addition, this suggests that careful attention to these parameters is critical for researchers who aim to understand foot and lower limb function.

Gastrocnemius medialis was not significantly affected by changes in muscle volume during braking and propulsion, except for during braking at the self-selected spontaneous velocity. This is contrary to our hypothesis that all three muscles would be impacted by variation in muscle volume. Gastrocnemius medialis and lateralis have been suggested to have separate, but complementary functions [30–32]. Their activation patterns have been demonstrated to vary with foot position and the two muscles differ in fiber length and pennation angle [30–32]. It has additionally been suggested that gastrocnemius medialis may play a more important role in propulsion during running [33], suggesting that the lack of consistent differences in muscle force may be indicative of a functional difference relative to the other triceps surae muscles during straight-path walking. Another possibility is that the algorithm used to estimate muscle forces in the model shifts force from soleus to gastrocnemius lateralis differently than it does to gastrocnemius medialis. More work investigating this possibility, including the potential impacts of muscle orientation, fiber typology, and limb/foot posture on muscle force allocation in the model, both of which remain unknown, would be beneficial.

### Other muscles/muscle groups

Variation in triceps surae muscle volume distribution also impacts maximum forces produced by other muscles, consistent with our hypothesis. The quadriceps muscle force during propulsion, for example, is relatively higher when gastrocnemius medialis and lateralis muscle volumes are relatively larger (as in the YHA configuration). Consistent with our prediction, the relatively larger quadriceps muscle force likely serves to counteract the larger gastrocnemius force, and thus the knee flexion moment. The foot evertors produce a relatively higher maximum force during propulsion when soleus is relatively larger, which was not predicted. The two evertors, fibularis longus and brevis, also work as plantarflexors, suggesting that they may be compensating for the relatively smaller gastrocnemius force at the ankle. It is also possible that these muscles are recruited to play a greater role in foot stabilization during walking when soleus muscle force is relatively higher.

There are also significant differences among the three configurations in maximum gluteus maximus, sartorius, hamstring, dorsiflexor, and other plantarflexor muscle forces at some velocities during the propulsive phase and for gluteus maximus and the hamstrings during the braking phase. The presence of significant differences in other, adjacent muscles/muscle groups to the triceps surae demonstrates that muscle parameters of a single muscle can impact the entire biomechanical chain. This suggests that researchers should not only pay careful attention to the parameters for the muscles they are interested in, but also those of other muscles in the musculoskeletal model.

## Conclusions

Muscle volume distribution substantially impacts muscle forces in musculoskeletal models. Including patient-specific muscle properties, in addition to current practices of utilizing individualized skeletal morphology and kinetic/kinematic data, may be important for patient-specific modeling. Our study demonstrates that this is particularly true for the muscles of interest, but also important for other muscles located further along the kinetic chain. For example, if muscle forces at the hip were of interest, it would also be important to consider the triceps surae muscle parameters.

This research also adds to the growing body of literature [e.g., 2] demonstrating that it is important to be careful with input parameters used in musculoskeletal models, as they can greatly impact the results. In making such considerations, it is important to evaluate the research question musculoskeletal modeling is being employed to address. For example, in the case of a human variation study, muscle volumes used by the model may be less critical, as they will affect all individuals in the same way. If values that are more reflective of reality are of interest, such as in patient-specific modeling, utilizing realistic muscle values for the individual is imperative.

## Supporting information

S1 Fig Muscle force profiles for soleus (A-B), gastrocnemius lateralis (C-D), and gastrocnemius medialis (E-F). Maximum muscle forces occur at similar percent step for all muscle configurations and individuals.
(PPTX)

S1 Data Maximum muscle forces for each subject at each velocity. (XLSX)
