# Supplementary material for: Implications of variability in triceps surae muscle volumes on peak lower limb muscle forces during human walking
Source: PLoS One. 2025 Mar 28;20(3):e0320516. doi: 10.1371/journal.pone.0320516 (PMC11952212; doi:10.1371/journal.pone.0320516)
Supplement: S1 Fig — Muscle force profiles for soleus (A-B), gastrocnemius lateralis (C-D), and gastrocnemius medialis (E-F). Maximum muscle forces occur at similar percent step for all muscle configurations and individuals. (PPTX) [file pone.0320516.s001.pptx]

## Slide 1
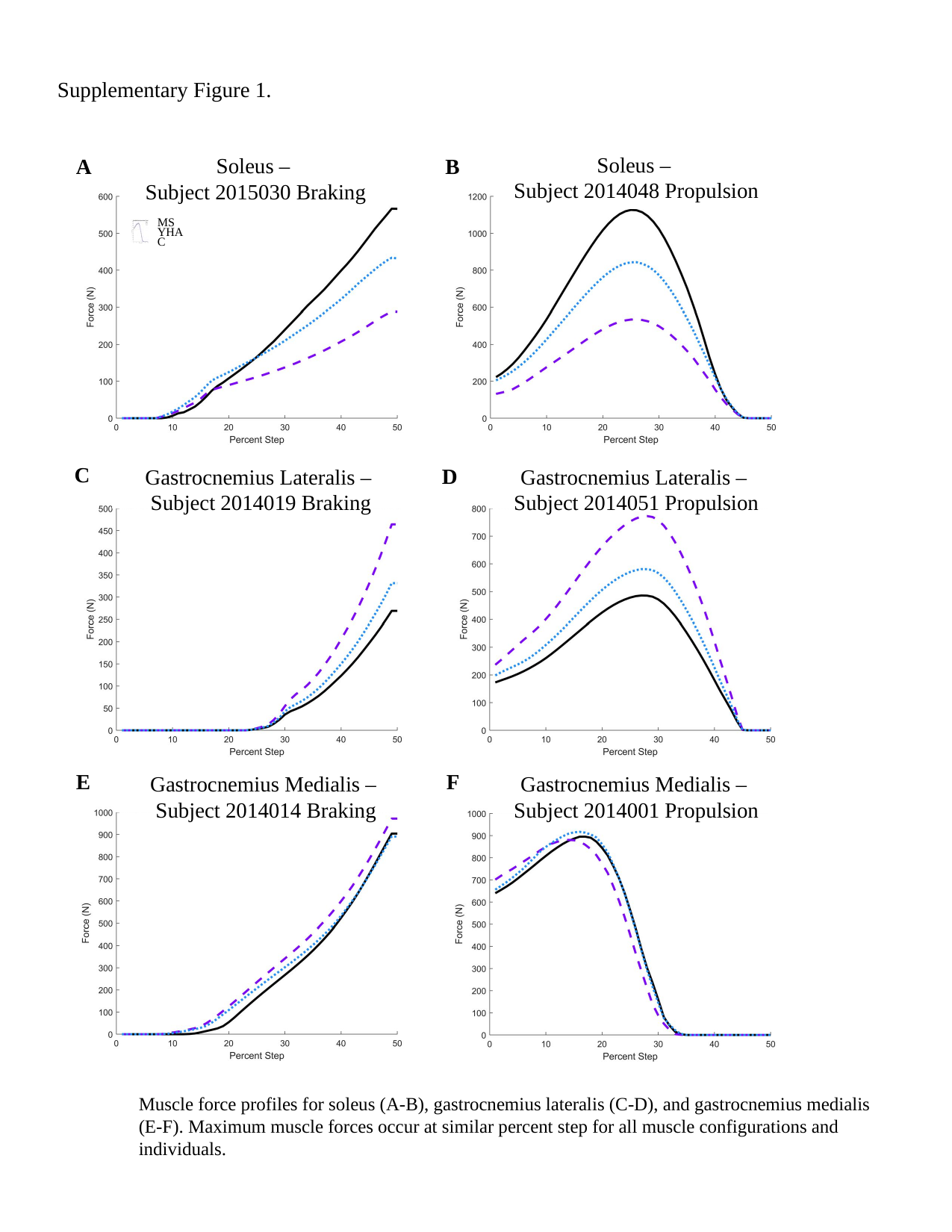

Supplementary Figure 1.
Soleus –
Subject 2014048 Propulsion
Soleus –
Subject 2015030 Braking
A
B
MS
YHA
C
C
D
Gastrocnemius Lateralis –
Subject 2014051 Propulsion
Gastrocnemius Lateralis –
Subject 2014019 Braking
E
F
Gastrocnemius Medialis –
Subject 2014001 Propulsion
Gastrocnemius Medialis –
Subject 2014014 Braking
Muscle force profiles for soleus (A-B), gastrocnemius lateralis (C-D), and gastrocnemius medialis (E-F). Maximum muscle forces occur at similar percent step for all muscle configurations and individuals.
